# Case report: disease mechanisms and medical management of calcium nephrolithiasis in rheumatologic diseases

**DOI:** 10.1186/s12894-023-01203-y

**Published:** 2023-03-23

**Authors:** Omar Osman, Susan Manzi, Mary Chester Wasko, Barbara A. Clark

**Affiliations:** Department of Medicine, Allegheny Health Network, 320 East North Ave, Pittsburgh, PA 15212 USA

**Keywords:** Case report, Nephrolithiasis, Renal tubular acidosis, Sjogrens syndrome, Sarcoidosis, Crohn’s disease, Vitamin D, Nephrocalcinosis, Kidney stones, Calcium metabolism, Osteoporosis

## Abstract

**Background:**

Nephrolithiasis as a feature of rheumatologic diseases is under recognized. Understanding presenting features, diagnostic testing is crucial to proper management.

**Case presentation:**

A 32 year old woman with a history of recurrent complicated nephrolithiasis presented to a rheumatologist for a several month history of fatigue, dry eyes, dry mouth, arthralgias. She had a positive double-stranded DNA, positive SSA and SSB antibodies. She was diagnosed with Systemic Lupus erythematosus (SLE) and Sjogren's syndrome and was started on mycophenalate mofetil. Of relevance was a visit to her local emergency room 4 years earlier with profound weakness with unexplained marked hypokalemia and a non-anion gap metabolic acidosis. Approximately one year after that episode she developed flank pain and nephrocalcinosis. She had multiple issues over the ensuing years with stones and infections on both sides. Interventions included extracorporeal shockwave lithotripsy as well as open lithotomy and eventual auto-transplantation of left kidney for recurrent ureteric stenosis. 24 h stone profile revealed marked hypocitraturia, normal urine calcium, normal urine oxalate and uric acid. She was treated with potassium citrate. Mycophenolate was eventually stopped due to recurrent urinary tract infections and she was started on Belimumab. Because of recurrent SLE flares, treatment was changed to Rituximab (every 6 months) with clinical and serologic improvement. Her kidney stone frequency gradually improved and no further interventions needed although she continued to require citrate repletion for hypocitraturia.

**Conclusions:**

Nephrolithiasis can be a prominent and even presenting feature in Sjogrens syndrome as well as other rheumatologic diseases. Prompt recognition and understanding disease mechanisms is important for best therapeutic interventions for kidney stone prevention as well as treatment of underlying bone mineral disease.

## Background

Rheumatologic conditions may not be initially considered in the differential if the presenting features are kidney stones or electrolyte abnormalities such as hypokalemia or metabolic acidosis [1–5]. However these can be the presenting manifestations of rheumatologic conditions that predominantly affect the renal tubules rather than the glomeruli [6–13].

### Case presentation

A 32 year old presented for a rheumatologic evaluation for a several month history of fatigue, dry eyes, dry mouth, weight loss, alopecia, arthralgias and dyspnea on exertion.

Review of medical records noted an unusual presentation to her local emergency room 4 years earlier with profound weakness where she was found to have marked hypokalemia with serum potassium 1.4 milliequivalents per liter. She also had a non-anion gap metabolic acidosis at that time with a serum bicarbonate of 12–14 milliequivalents per liter and a urine pH of 7–8. Magnesium level was normal. This episode was never explained but was attributed to possible “occult” diarrheal illness even though patient had denied diarrheal illness. The hypokalemia resolved with potassium chloride supplementation and she was maintained on potassium supplementation for some time subsequently.

**Fig. 1:**
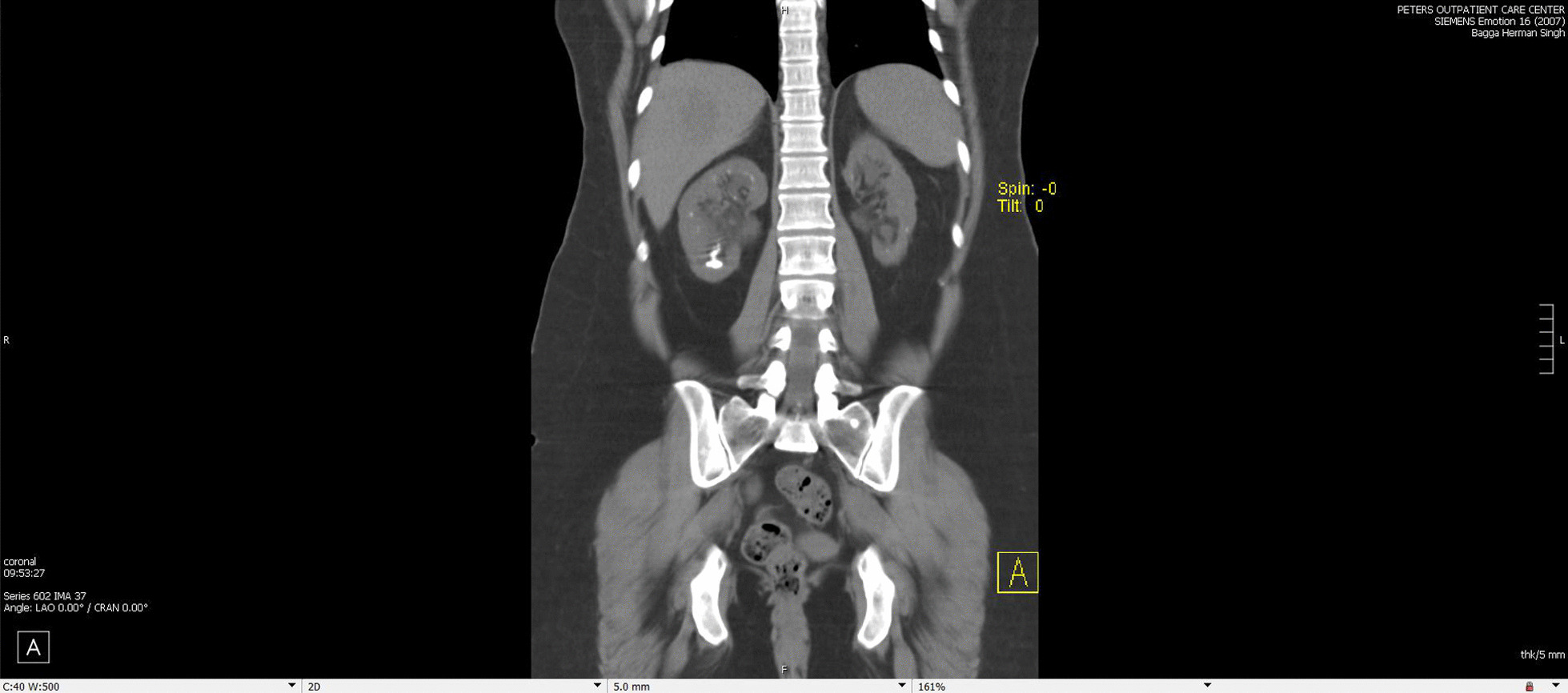
CT scan of patient in 2017 noting large right sided stones, nephrocalcinosis and left renal atrophy and dilated renal pelvis attributed to prior obstruction and surgical reimplantation of ureter done a few years earlier

**Fig. 2:**
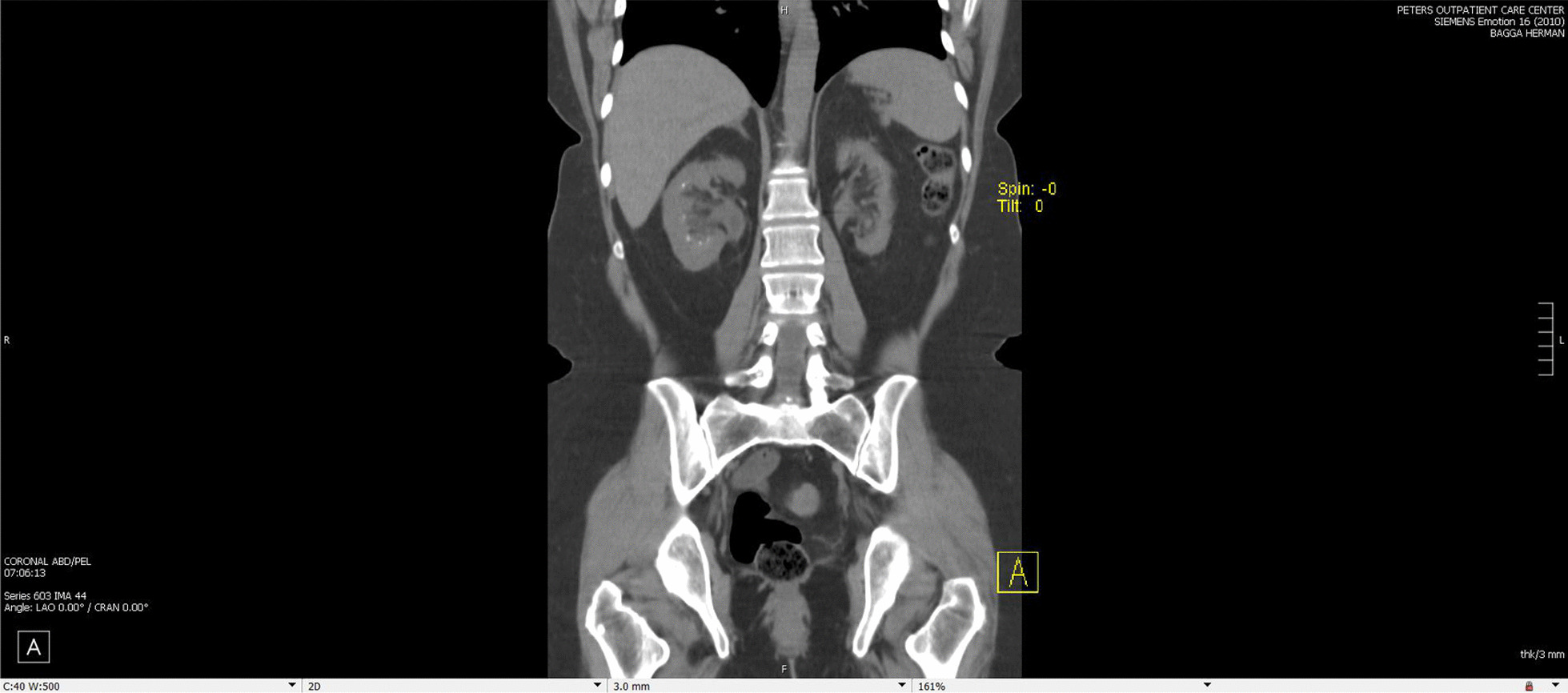
CT scan of patient from 2021 noting persistent nephrocalcinosis but no large stones and progressive left atrophy

Approximately one year after this episode she started having issues with flank pain and recurrent episodes of nephrolithiasis. CT scan noted nephrocalcinosis ( Figs. 1 and 2).

After her Rheumatology visit further work up included a positive ANA and positive double-stranded DNA (ds DNA) titer of 1:320. She also had a positive SSA and SSB. Rheumatoid factor was elevated at 28. C-ANCA was positive at 1:160 but specific PR 3 and MPO antibodies were negative. Serum protein electrophoresis revealed a polyclonal gammopathy. At that time her sedimentation rate was elevated at 50 mm/hr with a CRP that was mildly elevated at 2.2 mg/dL. She was found to have hypocomplementemia with a C3 level of 32 mg/dL and a C4 of 2.1 mg/dL. Microalbumin to creatinine ratio was within normal limits at 27 mg/g. Urinalysis revealed no proteinuria, but microscopic hematuria was present with 3–5 nondysmorphic red blood cells/high power field but no white blood cells or casts. A complete blood count showed a white blood cell count at that time was 4.7 k/mcL with a hemoglobin of 10.7 g/dL and a platelet count of 251 k/mcL. Serum creatinine at that time was 0.9 mg/dL with a potassium of 4.3 meq/L and a serum bicarbonate level of 18 meq/L.

Following the above lab testing along with her clinical symptomatology, she was given the diagnosis of Systemic Lupus erythematosus (SLE) and Sjogren's syndrome. Mycophenolate was initiated at a dose of 500 mg twice a day and she was also given a pulse dose of IV methylprednisolone and started on hydroxychloroquine. She had modest improvement in her symptoms and mycophenolate was increased to 1000 mg twice daily and was maintained on 8 mg of oral methylprednisolone per day with subsequent improvement in her symptoms. She also had improving sedimentation rate to 21 mm/hr and improving complement levels with C4 level of 13 mg/dL and a C3 level of 98 mg/dL. In addition, her double stranded DNA titer was declining. Hemoglobin had improved to 12.5 g/dL.

Because of the recurrent complicated nephrolithiasis, her rheumatologist also suggested a nephrology evaluation. Nephrology assessment included normal parathyroid hormone level ( 55 pg/ml, normal 11–68), 25 hydroxy Vitamin D was low at 18 ng/mL normal 30–100), 1,25 dihydroxy Vitamin D was normal range at 59 pg/ml, normal 20–79). Urinalysis revealed no proteinuria, some microscopic hematuria, variable amounts of pyuria without cellular casts or dysmorphic hematuria, occasional calcium oxalate crystals seen. 24 h stone profile revealed marked hypocitraturia (75 mg/d, normal > 550) with normal urine calcium (52 mg/d, normal less than 200), normal urine oxalate (21 mg/d, normal 20–40) and uric acid (459 mg/d, normal < 750). Kidney stone analysis noted predominantly calcium oxalate with a component of calcium phosphate. Bone density studies did not reveal any osteopenia or osteoporosis. After completion of 24 h urine, she was treated with potassium citrate (20 Meq BID) to replace her potassium chloride. Serum potassium levels remained within normal range with supplementation. Serum bicarbonate was intermittently low between 18 and21 meq/L, so additional sodium bicarbonate was added to her regimen. Vitamin D repletion was initiated. While there was initial concern about replacing Vitamin D because of nephrolithiasis, stone profiles documented low urine calcium which did not increase after Vitamin D repletion.

She had multiple issues over the ensuing years with stones and infections on both sides. Interventions included extracorporeal shockwave lithotripsy as well as open lithotomy and eventual auto-transplantation of left kidney for recurrent ureteric stenosis. She required over 20 interventions related to her renal stones. There were intermittent episodes of acute kidney injury related to obstruction and sometime associated infection. She developed some associated chronic kidney disease with serum creatinine rising from 0.8 mg/dl to 1.3 mg/dl over an 8 year span.

Follow up urine examinations noted pyuria, non-dysmorphic hematuria, no casts, protein quantification of 200–300 mg/ g creatinine with urine pH 6 to 6.5.

Mycophenolate was eventually stopped due to the recurrent urinary tract infections and she was started on Belimumab. This failed to control her SLE with worsening symptoms and decreasing C4 and rising ds-DNA, so mycophenolate was resumed and required intermittent escalation in steroid dosing. Because of recurrent SLE flares despite the mycophenolate, treatment was changed to Rituximab (every 6 months) with clinical and serologic improvement. Her kidney stone frequency and severity gradually improved over time- with less than one stone episode per year and no further interventions needed although she continued to require citrate repletion for hypocitraturia.

Patient comments: “" go ahead and use, my case. I think it is wonderful, as long as it helps somebody else, that would be great".

## Discussion and conclusions

### Kidney stone formation and nephrocalcinosis

Kidney stones develop when dissolved salts in the urine become solids. This occurs when salts exceed solubility ratios (Tables 1 and 2). This is called super saturation [14–20]. Four different variables can lead to supersaturation: (1) Low urine volume; (2) excess excretion of solutes such as calcium, oxalate, or uric acid; (3) lack of natural inhibitors of crystal formation, such as citrate or magnesium; and (4) urine pH. Urine volume is the main determinant of super saturation. In most kidney stone formers, urine calcium and urine oxalate concentrations are the main determinants of kidney stone formation and contribute to calcium oxalate super saturation and calcium oxalate stone formation [14–20]. Kidney stone formation is dependent not only on the urine solute concentrations but also on urine pH, with alkaline urine pH contributing to calcium phosphate super saturation and acidic urine pH contributing to calcium oxalate and uric acid supersaturation [18, 19]. Urine pH in turn is dependent on renal acid handling. [19, 20]. Urine contains many substances, most notably citrate, that hinder the formation of solid calcium oxalate and calcium phosphate. Urine citrate reduces super saturation by binding calcium and hence inhibits growth of calcium crystals. Hence, deficient levels of urine citric acid can contribute to stone formation irrespective of urine pH. Adequate amount of urinary magnesium is also believed to inhibit calcium oxalate formation by binding to urine oxalate.

**Table 1:** Factors that lead to stone formation

Properties that lead to stone formation
Low urine volume (leads to increased concentration of available solutes)
Excess excretion of solutes (calcium, oxalate, uric acid)
Lack of crystal forming inhibitors (citrate, magnesium)
Urine pH changes (Alkaline leads to calcium phosphate supersaturation, Acidic leads to calcium oxalate and uric acid supersaturation)

**Table 2:** Recommended lab workup for patients with recurrent kidney stone

Laboratory workup for recurrent kidney stones
Serum electrolytes (helps to identify underlying hyperparathyroidism, hyperuricemia and RTA)
Urine composition, two separate 24 h urine collections with patients on their usual diet, fluid intake and physical activity (assess urine volume, calcium, uric acid, citrate, oxalate, creatinine, pH and sodium
Radiography if not yet performed (degree of radiopacity may suggest type of stone present)

Calcium oxalate stone formation correlates directly with urine calcium and /or oxalate level and indirectly with urine volume, pH and citrate. Normal urine citrate level is greater than 350–400 mg/day. [20]. Conventional upper limits of urine calcium for women is about 250 mg per day and about 300 mg per day for men [20]. Patients who exceed this level of urine calcium require further diagnostic evaluation. This should include assessment of serum calcium levels, parathyroid hormone levels, and evaluation of 1, 25 dihydroxy vitamin-D (calcitriol) levels [20, 21]. The upper limit for normal oxalate is around 40 mg/day. Patients with excess oxalate require a careful assessment for fat malabsorption as well as careful dietary history for possible diet excess (including tea, spinach, certain nuts, wheat germ, or vitamin C). (Table 2).

Patients may present with nephrocalcinosis and/or nephrolithiasis. While these are distinct entities, there is an underlying common mechanism. Nephrocalcinosis refers to the diffuse precipitation of calcium salts within the tubular epithelium and interstitial tissue of the kidney [20]. This generally involves the medulla and can be detectable by ultrasound or CT scan. Nephrolithiasis refers to kidney stones that are larger and generally visible on plain radiographs, ultrasounds and CT scans [20]. Both nephrolithiasis and nephrocalcinosis frequently evolve from the metabolic abnormalities described above. Those abnormalities can include hypercalciuria, hyperoxalauria or hypocitraturia and/or defects in urinary acidification.

The vast majority of calcium oxalate stone formers do not have any systemic disease and are described as idiopathic stone formers. The defect is often hypercalciuria, although the genetic mechanisms can vary [21, 22]. Some stone formers are discovered to have primary hyperparathyroidism where PTH excess leads to increased bone resorption as well as increased synthesis of calcitriol which increases intestinal calcium absorption which in turn leads to increased filtered load of calcium at the kidney. Others may have dietary issues or underlying bowel condition. Malabsorption of fatty acids or bile salts such as in Crohn’s disease or after gastric bypass leads to increased colonic intestinal oxalate absorption that contributes to excess urine oxalate [20]. Those with primary bowel disorders are labeled as having enteric hyperoxaluria. Those patients found to have excess calcitriol levels need a careful consideration for the possibility of underlying sarcoidosis, although this can sometimes be the phenotype of genetic hypercalciuria.

### Nephrolithiasis and sjogrens

While hypercalciuria is the driving factor in most idiopathic kidney stone formers, it may not be the driving factor in patients with rheumatologic diseases (other than sarcoidosis) [23, 24]. Patients with autoimmune conditions can develop antibodies directed against tubular transporters of potassium and hydrogen ion that can first present as marked hypokalemia and non-anion gap metabolic acidosis [11]. Over time this leads to nephrocalcinosis based on alterations in urine pH. However some of these patients are actually also discovered to develop hypercalciuria, for mechanisms discussed below.

**Fig. 3:**
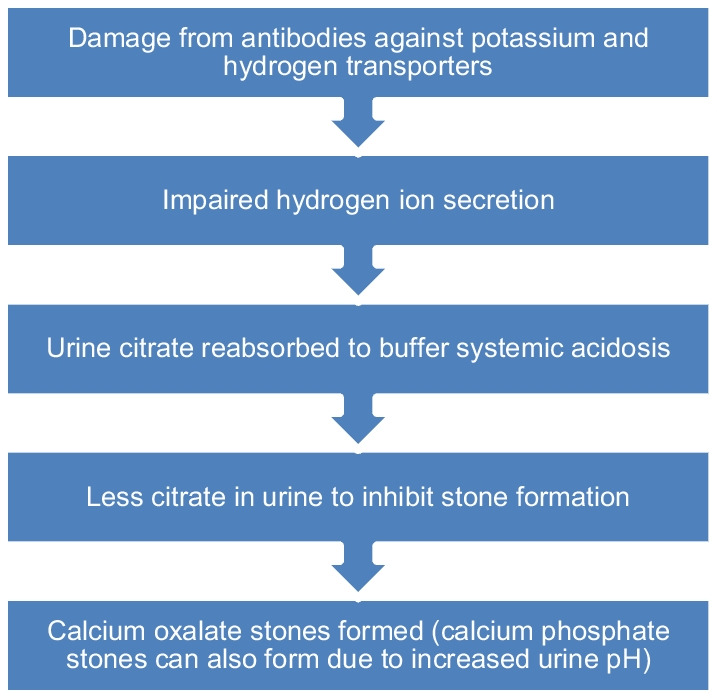
Sjogrens disease mechanism leading to stone formation

The exact mechanisms of development of these tubular abnormalities in patients with Sjogren's or other autoimmune conditions is not well understood, but most reported cases have had antibodies in the systemic circulation typical of Sjogren's syndrome with positive antibodies to SSA and SSB [1–3, 11, 25, 26] (Fig. 3). As there can be often overlap and coexistence of Sjogren’s with systemic lupus erythematosis many patients with these type of stones can have features of both disorders, as was the case in our patient. Renal biopsy is rarely performed, but in cases where done, showed tubular interstitial lymphoplasmacytic infiltrates as well as fibrosis [6, 10]. Typically, urine pH is 6.5 or above and most start out with hypocitraturia rather than hypercalciuria [25, 26]. The kidney stones are composed of predominately calcium oxalate but can be mixed with calcium phosphate present as well.

There have been numerous case reports or case series outlining the occurrence of nephrolithiasis or nephrocalcinosis with Sjogren’s disease. There have been at least 15 publications since 1991 (27–41). The incidence of nephrolithiasis or nephrocalcinosis in case series of renal manifestations of Sjogren’s has been reported to range from 7 to 40% [27–29, 31, 41]. Some cases have even noted nephrocalcinosis as the presenting manifestation [40]. The unifying feature in all cases is renal tubular acidosis. Some cases have noted coexistence with other rheumatologic diseases such as lupus [37].

The renal tubular acidosis is described as type 1 RTA that is an acquired defect [25, 26]. The cause of the hypocitraturia is due to increased tubular re-absorption of the filtered citrate in response to the systemic acidosis. Hypokalemia is often the original presenting feature presumably due to antibody mediated impairment of potassium reabsorption [9]. Some cases are so severe that they present with flaccid paralysis related to the hypokalemia [13]. The hypokalemia is often self-limited while the acidosis persists and is later followed by nephrocalcinosis and nephrolithiasis as well as bone demineralization [26–28].

There are several key ion transporter proteins throughout the kidney tubules important in hydrogen ion secretion and bicarbonate regeneration. These include the chloride- bicarbonate exchanger (AE1), Hydrogen ATPase, intracellular carbonic anhydrase, and Potassium ATPase in the intercalated cells in the distal tubule [25]. These can be hereditary conditions or can be acquired. One of the most common acquired forms of defects in one of these transporters is Sjogren's syndrome [25, 26]. Defects in any of these areas are associated with type 1 distal renal tubular acidosis. There have been reports of autoantibodies to carbonic anhydrase and one report that involved a kidney biopsy showed the absence of hydrogen ATPase in the distal tubules of a patient with Sjogren's syndrome and distal RTA [26]. However this does not rule out antibody mediated defects in some of the other transporters. The original defect may be impaired hydrogen secretion. When there is inability to secrete hydrogen ion into the tubular lumen this results in significant hypokalemia, as potassium is now the only cation that can be exchanged in the distal tubule when sodium is reabsorbed. This leads to renal potassium wasting. In turn there is development of nephrocalcinosis and nephrolithiasis. This is in part related to hypocitraturia, as citric acid is avidly re-absorbed in the proximal tubule to help buffer systemic acidosis. The absence of citrate in the urine provides nothing to stabilize and prevent calcium oxalate precipitation.

### Connection to bone disease

In addition, over time, there can be development of hypercalciuria and hyperphosphaturia as both calcium and phosphorus are leeched from the bones, as the bone serves as the major buffer source for systemic acidosis [26, 44–46]. In addition, the alkaline urine pH further contributes to calcium phosphate precipitation [14–17]. This chronic acidosis hence contributes to bone dissolution and osteomalacia.

Calcium carbonate stores in bone are the main source of available bicarbonate buffer during systemic acidosis. Acid induced dissolution of bone apatite leads to osteomalacia [44–46]. Furthermore, chronic urinary calcium losses can also lead to bone demineralization with resultant decreased bone mineral density and osteoporosis [43–46]. This can have serious implications for bone health especially as most patients are also at increased risk of premature osteoporosis due to corticosteroid exposure.

### Treatment: sjogrens associated acquired RTA

Disease remitting agents for Sjogrens may be able to decrease the antibody production that had triggered the initial defect if the process has not already led to tubular damage and renal tubular fibrosis [3]. For the nephrolithiasis, key treatment strategies include replacement of urinary citrate with potassium citrate and correction of systemic acidosis with oral potassium citrate and/or sodium bicarbonate [1–3] (Tables 2 and 3). In most cases potassium citrate is preferred as sodium bicarbonate or sodium citrate repletion can trigger increase urine calcium excretion [14–17]. Follow up of 24 h urine values a few months after initiating or adjusting therapy are appropriate to ensure citrate is replete and to assess for the possibility of co- occurrence of hypercalciuria that might also need to be treated with agents such as thiazide diuretics to reabsorb urine calcium [14–16]. If thiazide diuretics are used, additional potassium supplementation may also be required to offset urinary potassium losses. It is also important to address the underlying disease process.

**Table 3:** Treatment options for stones in each condition other than treating underlying condition

	Potassium citrate	Sodium bicarbonate/citrate	Thiazides	Vitamin D	Dietary changes	Others
Sjogren’s	Repletes potassium and corrects acidosis	Can be used to correct acidosis, may trigger increase urine calcium excretion	If there is underlying hypercalciuria (potassium supplementation may be needed)			
Sarcoidosis			Avoid (can aggravate underlying condition)	Avoid (can aggravate underlying condition)		Ketoconazole (off label use)
IBD		Can be considered in ileostomy patients with bicarbonate loss			Low oxalate, low fat diet. Increase citrus fruits	Calcium supplements, pyridoxine, magnesium

### Nephrolithiasis and sarcoidosis

**Fig. 4:**
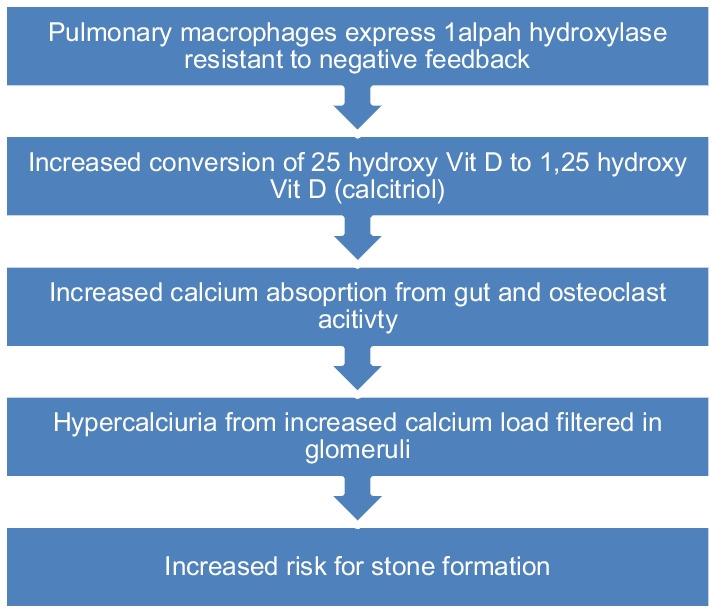
Sarcoidosis disease mechanism leading to stone formation

Renal calculi have been reported to occur in about 10–20% of patients with chronic sarcoidosis. Other studies report, the incidence of nephrolithiasis is variable with a range ranged between 6 and 12% [23, 24]. There appears to be a predilection for stones in Caucasian male with peak incidence between 40 to 60 years of age. The pathophysiology of nephrocalcinosis appears to revolve primarily around serum calcium and vitamin-D metabolism (Fig. 4). The activated pulmonary macrophages in sarcoid granulomas express 1 alpha hydroxylase which is resistant to the normal negative feedback mechanism when hypercalcemia develops. As a result of this autonomous activity, 25 hydroxy Vitamin D is metabolized into the much more biologically active calcitriol (1,25 dihydroxy Vitamin D). Increased amounts of Calcitriol then lead to increased intestinal absorption of calcium in addition to activating osteoclasts. This leads to hypercalciuria from the increased calcium load filtered at the level of glomeruli along with suppression of PTH which would normally increase tubular reabsorption of calcium.

In about 1% of cases, nephrolithiasis is the initial manifestation of sarcoidosis [2, 23]. An additional 2.7% of patients were found to have asymptomatic stones when sarcoidosis was diagnosed otherwise. Less than 5% with sarcoidosis will developed nephrocalcinosis, but can lead to progressive chronic kidney disease [23].

### Treatment: Sarcoidosis

Treatment of the nephrolithiasis in sarcoidosis involves treatment of the underlying disease with corticosteroids along with adequate fluid intake [22, 23]. Refractory patients could be considered for treatment of medications that inhibit cytochrome P 450, as alpha hydroxylase excess activity in the macrophages from granulomas is a cytochrome p 450 dependent enzyme. This is somewhat analogous to that in some genetic hypercalciuric stone forms, where one of the enzyme defects involves CYP24A1. [47–49] (Fig. 4). However, these are off label use of medications (such as ketoconazole) and can have other side effects and are not part of the usual therapeutic approach. Vitamin D repletion is not appropriate in these patients as the defect is excess endogenous conversion to the active form, calcitriol [47–49]. While most idiopathic hypercalciuric patients benefit from treatment with thiazide diuretics to reabsorb urine calcium, this strategy is not typically used in sarcoidosis as this can aggravate underlying hypercalcemia [14–16].

### Nephrolithiasis and inflammatory bowel disease (IBD)

**Fig. 5:**
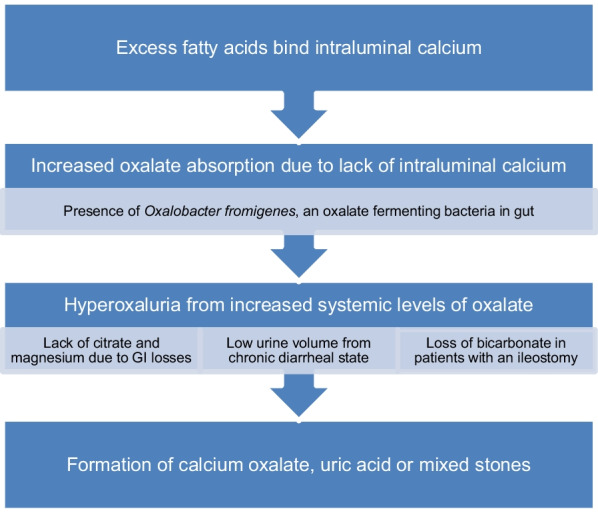
IBD disease mechanism leading to stone formation

Crohn’s disease and Ulcerative Colitis are systemic diseases that can have extraintestinal manifestations in 6 to 20% of cases [50]. These manifestations often lead to rheumatologic care and can include uveitis, iritis, ankylosing spondylitis, pyoderma gangrenosum, and erythema nodosum [50]. Nephrolithiasis is also more common in patients with IBD than in the general population, as high as 9–18%, especially those with Crohn’s disease (51.52) ( Fig. 5). The types of stones can be calcium oxalate, uric acid or mixed stones. In IBD, hypercalciuria itself is rare, unless an underlying genetic defect or in cases of excess Vitamin D repletion [37]. Risk factors for kidney stone formation in Crohn’s disease include the following: (1) malabsorption of fatty acids/bile salts. Excess fatty acid bile salt presence binds intraluminal intestinal calcium. This leads to less insoluble calcium excretion in the stool and hence less calcium to bind oxalate. This in turn leads to higher oxalate absorption from the colon and increase in oxalate in the systemic circulation and hence the urine. This mechanism does not occur if colon is surgically absent. (2) A second important mechanism is lack of natural inhibitors of stone formation- less urinary citrate and magnesium due to gastrointestinal losses. (3) Chronic diarrheal illness also leads to chronic volume depletion and low urine volume with increased risk of super saturation of all salts. (4) In patients with ileostomy there are large losses of bicarbonate that leads to chronic systemic acidosis and acidic urine pH. This acidic urine pH and low urine volume increases risk of uric acid and calcium oxalate stone risk. (5) Finally, there may also be lower levels of the oxalate fermenting bacteria, *Oxalobacter Fromigenes*, contributing to increase in intestinal oxalate availability [51–54]. Patients with ulcerative colitis have increased risk of uric acid and calcium oxalate stones, especially with ileostomy, due to low urine volume, low urine pH but do not have the increase urine oxalate typical of Crohn’s patients [51–54].

### Treatment of stones in IBD

Mainstay of treatment in all is to increase fluid intake as much as possible to achieve urine volume above 2 L. In those with enteric hyperoxaluria a low oxalate diet is important. Low oxalate diet involves avoiding spinach, rhubarb, almonds, cashews, peanuts, cocoa powder, bran cereal, wheat germ, Swiss chard, beets and vitamin C [51, 55, 56]. Low fat diet is also appropriate to decrease intestinal calcium binding. In those with hyperoxaluria, it is also beneficial to use calcium supplements with meals to bind oxalate in the gut and prevent absorption [51, 55, 56]. Dietary calcium of 800–1200 mg daily is suggested [5–51, 51–58]. Use of pyridoxine (vitamin B6) may also be associated with lower urine levels of oxalate and decreased risk of stone formation [56, 59]. The enzyme (alanine glycoxylate aminotransferase) that converts systemic glycoxylate to glycine instead of oxalate is dependent on pyridoxine as a cofactor. Hence pyridoxine deficiency can be associated with increase conversion of glycoxylate to oxalate. Recommended pyridoxine dose is approximately 100 mg daily [56, 59]. Magnesium repletion is also suggested in cases of magnesium deficiency as magnesium can also serve to bind urine oxalate and inhibit calcium oxalate formation [52]. In those with low urine citrate, increased intake of fruits and vegetables (excluding those with high oxalate content) especially citrus fruits is beneficial. Use of citrate or alkalinizing agents (such as bicarbonate) to achieve a target urine pH of greater than 6.5 [51, 53, 55]. Uric acid stones may even dissolve with alkalinzation of the urine [14–17]. Use of xanthine oxidase inhibitors such as allopurinol or febuxostat are seldom indicated unless there is associated condition such as gout where there is overproduction and significant hyperuricosuria (greater than 900 mg uric acid per day) or if uric acid stones recur despite the other maneuvers to increase urine pH and volume [14–17].

### Treatment of bone mineral axis

It is appropriate to assess bone mineral density. While treatment with calcium supplements can potentially aggravate the stone risk in some cases [58], due to increased risk of GI absorption, increase in dietary calcium is appropriate to 800 to 1200 mg daily [55–58]. In fact, diets low in calcium can actually increase stone risk by increasing risk of excess urine oxalate, as intestinal calcium helps to bind oxalate in the intestine and prevent excess oxalate absorption [14–17, 58]. Furthermore, in cases of fat malabsorption such as with Crohn’s disease, calcium supplements with meals will increase oxalate binding with calcium in the intestine to prevent excess oxalate absorption. Most patients will benefit from vitamin D repletion as long as there is not an associated genetic or acquired predisposition to idiopathic hypercalciuria, as in sarcoidosis. If vitamin D is supplemented, it is prudent to reassess urinary calcium excretion with either 24 h urine or random urine calcium to creatinine ratios. If patients are found to have hypercalciuria, treatment with a thiazide type diuretic may be used to trigger increased renal reabsorption of calcium [14–17]. As this can trigger urinary potassium loss, this may require increase use of potassium supplementation. Other mechanisms to stabilize bone may include bisphosphonate use (avoid if GFR < 30) or denosumab [60–62]. In severe cases, teriparatide may be considered if not contraindicated by degree of kidney failure [62].

## Conclusion

As in our case illustration, Sjogren's disease can present with severe non-anion gap metabolic acidosis related to a defect in one of the hydrogen transport mechanisms in the distal tubule, presumed to be antibody mediated. This then leads to obligate renal potassium wasting as only potassium can be exchanged in the distal tubule when sodium is reabsorbed. These patients can present with profound muscle weakness due to hypokalemia and also have non anion gap metabolic acidosis, as in our patient. The cause of the hypokalemia and acidosis can remain elusive as other overt manifestations of Sjogrens may not be clinically apparent for months to years. These changes will eventually lead to nephrocalcinosis and nephrolithiasis due to hypocitraturia and inability to stabilize urinary calcium which leads to calcium oxalate supersaturation. Over time, hypercalciuria and hyperphosphaturia can develop as well as potential for bone to become demineralized as a source of alkali to offset the systemic acidosis. The alkaline urine pH further contributes to calcium phosphate precipitation. In addition over time, this systemic acidosis and bone demineralization can result in osteoporosis and increased fracture risk.

Other rheumatologic conditions with important potential manifestations of nephrolithiasis include the vitamin D mediated hypercalciuria in sarcoidosis and the low urine volume, low urine pH, hypocitraturia, and potential hyperoxaluria in IBD with increased risk of both calcium and uric acid stones.

In summary, patients presenting with recurrent nephrolithiasis can have underlying rheumatologic conditions that should be considered in the evaluation. In the case of Sjogrens (which can coexist with systemic lupus), the presentation can include hypokalemia and metabolic acidosis and is characterized by hypocitraturia and eventually hypercalciuria. In the case of sarcoidosis the presentation can include hypercalcemia and is associated with hypercalciuria. In the case of IBD there is often associated hypovolemia, hypocitraturia, hyperuricosuria or hyperoxaluria when there is significant malabsorption (when there is an intact colon). Prompt recognition, assessment of risks and implementation of treatment could potentially serve as prevention of these sequelae.

## Data Availability

Available in the electronic medical records (EPIC).

## Abbreviations

SLE: Systemic Lupus Erythematosis
SSA/SSB: Anti- sjogrens- syndrome- related antigen A and B autoantibodies
IBD: Inflammatory bowel disease
RTA: Renal tubular acidosis
ds DNA: Double stranded deoxyribonucleic acid
ANA: Antinuclear antibody
pr3: Proteinase 3
mpo: Myeloperoxidase
C4: Complement 4
C3: Complement 3
AE-1: Anion exchanger-1

## References

[1] Ramos-Casals M, Tzioufas AG, Font J (2005). Primary Sjögren's syndrome: new clinical and therapeutic concepts. Ann Rheum Dis.

[2] Tan EM, Cohen AS, Fries JF (1982). The 1982 revised criteria for the classification of systemic lupus erythematosus. Arthritis Rheum.

[3] Mariette X, Criswell LA (2018). Primary sjogrens syndrome. NEJM.

[4] Petri M, Magder L (2004). Classification criteria for systemic lupus erythematosus: a review. Lupus.

[5] Weening JJ, D'agati VD, Schwartz MM, Seshan SV, Alpers CE, Appel GB, Balow JE, Bruijn JA, Cook T, Ferrario F, Fogo AB (2004). The classification of glomerulonephritis in systemic lupus erythematosus revisited. Kidney Int.

[6] Maripuri S, Grande JP, Osborn TG, Fervenza FC, Matteson EL, Donadio JV, Hogan MC (2009). Renal involvement in primary sjogren’s syndrome: a clinicopathologic study. Clin J Am Soc Nephrol.

[7] Toosi TD, Naderi N, Movassaghi S, Seradj MH, Khalvat A, Shahbazi F. Secondary sjogren's syndrome presenting with hypokalemic periodic paralysis .Oxford Medical Case Reports 2014; 1(8): 135–137. 10.1093/omcr/omu052 PMC4369992 25988057

[8] Dowd JE, Lipsky PE (1993). Sjogren’s syndrome presenting as hypokalemic periodic paralysis. Arthritis Rheum.

[9] Garza-Alpirez A, Arana-Guajardo AC, Esquivel-Valerio A, Villarreal-Alarcón MA, Galarza-Delgado DA. Hypokalemic paralysis due to primary sjögren syndrome: case report and review of the literature case reports in rheumatology. Volume 2017; 2017: 7509238 10.1155/2017/7509238 PMC5556603 28835864

[10] Shioji R, Furuama T, Onodera S, Saito H, Ito H, Sasai Y (1970). Sjogrens syndrome and renal tubular acidosis. Am J Med.

[11] Bastani B, Haragism L, Gluck S, Siamopoulos KC (1995). Lack of H-ATPase in distal nephron causing hypokalemic distal RTA in a patient with SjogrenSyndrome. Nephrol Dial Transplant.

[12] Kawashia M, Amano T, Morita Y, Yamanura M, Makino H (2006). Hypokalemic paralysis and osteomalacia secondary to renal tubular acidosis in a case with primary sjogren syndrome. Mod Rheumatol.

[13] Goroshi M, Khare S, Jamale T, Shah NS (2017). Primary sjogren's syndrome presenting as hypokalemic paralysis: a case series. J Postgraduate Med.

[14] Sakhaee K, Maalouf NM, Sinnott B (2012). Kidney stones 2012: pathogenesis, diagnosis, and management. J Clin Endocrinol Metab.

[15] Worcester EM, Coe FL (2008). Nephrolithiasis. Prim Care.

[16] Coe FL, Evan A, Worcester E (2005). Kidney stone disease. J Clin Invest.

[17] Coe FL, Parks JH, Evan AP, Worcester E, Alpern RJ, Hebert SC (2007). Pathogenesis and treatment of nephrolithiasis. Seldin and Giebisch's The Kidney.

[18] Kumar V, Lieske JC (2006). Protein regulation of intrarenal crystallization. Curr Opin Nephrol Hypertens.

[19] Evan AP, Lingeman J, Coe F, Shao Y, Miller N, Matlaga B (2007). Renal histopathology of stone-forming patients with distal renal tubular acidosis. Kidney Int.

[20] Sherrard DJ (1983). Metabolic causes of nephrolithiasis. Western J Med.

[21] Moe OW, Bonny O (2005). Genetic hypercalciuria. J Ame Soc Nephrol.

[22] Sayer JA (2017). Progress in understanding the genetics of calcium containing nephrolithiasis. JASN.

[23] Rizzato G, Fraioli P, Montemurro L (1995). Nephrolithiasis as a presenting feature of chronic sarcoidosis. Thorax.

[24] Tebben PJ, Singh RJ, Kumar R (2016). Vitamin D mediated hypercalcemia: mechanisms, diagnosis and management. Endocrine Rev.

[25] Kino-Ohsaki J, Nishimari I, Morita M, Okazaki K, Yamamoto Y, Onishi S, Hollingsworth MA (1996). Serum autoantibodies to carbonic anhydrase I and II in patient with idiopathic chronic pancreatitis and Sjogren syndrome. Gastroenterology.

[26] Sandhya P, Danda P, Rajanatham S, Thomas N (2014). Sjogrens, RTA and oteomalacia, an Asian Indian series. Open Rheumatol J.

[27] Arman F, Shakeri H, Nobakht N, Rastogi A, Kamgar M (2017). A case of kidney I involvement in primary sjogren syndrome. Am J Case Rep.

[28] Fujisawa,Y. SuzukT. Zoshima,S, Hara S, Ito K, Mizushima I,,Fujii H, Kawano M. High frequency of kidney stones and or nephrocalcinosis in primary Sjogren syndrome might accelerate chronic renal dysfunction due to tubular interstitial disease. And eles of the rheumatic diseases, 2020; 79: FR 1066

[29] Bossini N, Savoldi S, Franceschini F, Mombelloni S, Baronio M, Cavazzana I, Viola BF, Valzorio B, Mazzucchelli C, Cattaneo R, Scolari F (2001). Clinical and morphological features of kidney involvement in primary Sjögren's syndrome. Nephrol Dialysis Transp.

[30] Schilcher G, Schwarz C, Hermann J (2017). Successful treatment of renal tubular acidosis and recurrent secondary struvite kidney stones with rituximab in a patient with primary Sjögren’s syndrome. Rheumatology.

[31] Nikolova M, Ivanov G, Makova G, Iliev A, Hristova M, Georgieva I, Gencheva Z, Nikolova R, G. Sheinkova, Vlaho Y**+**, Alexiev E,, Petkov K, Boneva T, Liubomirova M, Krasteva R, Bogo B, Todorov T, Elenkova E. Sjogren’s syndrome and the kidney. Archiv Immunol Allergy 2018; 1: 20–24

[32] Basok AB, Haviv YS, Rogachev B, Vorobiov M (2021). Renal tubular acidosis type 1 with prominent hypokalemia and nephrolithiasis as a presentation of Sjogren’s /systemic lupus erythematosus disease. Case Rep Nephrology Dial.

[33] Mbengue M, Ouanekpone C, Diagne S, Niang A (2021). From hypokalemic crisis to sjogren’s syndrome: a case report and literature review. Case Reports Nephrol Dialysis.

[34] Linan LMJ, Montero SAR, Marenco de la Fuente JL. Nephrocalcinosis at patient with rheumatoid arthritis and secondary Sjogren’s syndrome. J Reumae 2017; 2: 005 10.1016/j.reuma.2017.02.006 28359765

[35] Martin W, Shye M. 35-year-old female with Sjogren syndrome and chronic kidney disease. Proceedings UCLA health 2020; 24

[36] Aguilera S, Lopez R, Valdivieso A (1996). Distal renal tubular acidosis and nephrolithiasis in 3 cases of primary Sjögren syndrome. Rev Med Chil.

[37] Zanuto AC, Bueno T, Delfino VD, Mocelin AJ (2012). Nephrocalcinosis in a patient with Sjögren's syndrome/systemic lupus erythematosus. Rev Assoc Med Bras.

[38] Moutsopoulus HM, Cledes J, Skopouli M, Eliasf M, Youinou P. Nephrocalcinosis in Sjogren syndrome: a late manifestation of renal tubular acidosis. J Intern Med; 1991, 230(2): 187–191. 10.1111/j.1365-2796.1991.tb00429.x 1865172

[39] Rajput R, Sehgal A, Jain D, Sen R, Saini O (2012). Nephrocalcinosis: a rare presenting manifestation of primary Sjögren’s syndrome. Modern Rheumatol.

[40] Dworsky Z, Bakhoun C, Chiraseveenuprapund P, Khare M, Nourbakhsh ND (2019). Sjogren’s syndrome presenting with distal renal tubular acidosis in adolescent. Biomed J Sci Techn Res.

[41] Aasarød K, Haga HJ, Berg KJ, Hammerstrøm J, Jørstad S (2000). Renal involvement in primary Sjögren's syndrome. QJM.

[42] Hajjaj-Hassouni N, Guedira N, Lazrak N, Harsouni F, Filali A, Mansouri A, Balafrej L (1995). Osteomalacia as presenting manifestation of Sjogren Syndrome. Rev Rheum Engl Ed.

[43] Cherie E, Ben Hassine L, Kaoueche Z, Khalfallah N (2013). Osteomalacia as inaugural manifestation of sjogrens syndrome. BMJ Case Rep..

[44] Kreiger NS, Bushinsky DA (2013). The relation between bone and stone formation. Calcif Tissue Int.

[45] Krieger NS, Frick KK, Bushinsky DA (2004). Mechanism of acid-induced bone resorption. Curr Opinion Nephrol Hypertension.

[46] Arrabal-Polo MA, Cano-Garcia Mdel C, Canales BK, Arabal- MM (2014). calcium nephrolithiasis in bone demineralization: pathophysiology diagnosis and management. Current opinion Urology.

[47] Jiráčková J, Hyšpler R, Alkanderi S, Pavlíková L, Palicka V, Sayer JA (2019). Novel CYP24A1 mutation in a young male patient with nephrolithiasis: case report. Kidney Blood Press Res.

[48] Sayers J, Hynes AM, Sricvstava S, Dowen F, Quinton R, Datta HR (2015). Successful treatment of hypercalcemia associated with a CYP24A1 mutation with fluconazole. Clin Kidney J.

[49] Ketha H, Singh RJ, Grebe SK, Bergstralh EJ, Rule AD, Lieske JC. Kumar R**.** Altered calcium and vitamin D homeostasis in first-time calcium kidney stone-formers. PLos ONE (2015); 10(9):e0137350. 10.1371/journal.pone.0137350 PMC4558059 26332888

[50] Bernstein LN, Blanchard JF, Rawsthorne P, Yu N (2001). The prevalence of extraintestinal disease in inflammatory bowel disease: a population based study. Am J Gastroenterol.

[51] Worcester EM (2002). Stones from bowel disease. Endocrinol Metab Clin North AM.

[52] Bianchi L, Gaiani F, Bizzarri B, Minelli R, Cortegosov P, Leandro G, DiMario F, Luigi de Angelis G, Roberto C. Renal lithiasis and inflammatory bowel disease, an update on pediatric population. Acta Biomed 2018; 89 (9) 76–80. 10.23750/abm.v89i9-S.7908 PMC6502195 30561398

[53] Trinchieri A, Lizzano R, Catelnuvo C, Zanetti G, Pisani E (2002). Urinary patterns of patients with renal stones associated with chronic inflammatory bowel disease. Arch Ital Urol Androl.

[54] McConnell N, Campbell S, Gillander I, Rolton H, Danesh B (2002). Risk factors for development of renal stones in inflammatory bowel disease. BJU Int.

[55] Prezioso D, Strazzalo P, Lotti T, Bianchi G (2015). CLU working group. Dietary treatment of urinary risk factors for renal stones. Arch Ital Urol.

[56] Gul Z, Monga M (2014). Medical and dietary therapy for kidney stone prevention. Korean J Urol.

[57] Curhan GC, Willett W, Rimm EB, Stampfer MJ (1993). A prospective study of dietary calcium and other nutrients and the risk of symptomatic kidney stones. N Engl J Med.

[58] Curhan GC, Willett WC, Speizer FE, Spiegelman D, Stampfer MJ (1997). Comparison of dietary calcium with supplemental calcium and other nutrients as factors affecting the risk for kidney stones in women. Ann Int Med.

[59] Curhan GC, Willett WV, Speizer FE, Stampfer MJ (1999). Intake of Vitamin B6 and C and the risk of kidney stone in women. J Am Soc Nephrol.

[60] Bianchi G, Guisti A, Piol G, Barone A, Palummeri E, Girasole G (2010). bisphosphonates in management of idiopathic hypercalciuria associated with osteoporosis. Therapeutic Adv Musculoskeletal Disease.

[61] Frick KK, Krieger NS, Bushinsky DA (2015). Modeling hypercalciuria in the genetic hypercalciuric stone-forming rat. Curr Opinion Nephrology Hyper.

[62] Cosman F (2014). Anabolic and antiresorptive therapy for osteoporosis: combination and sequential approaches. Curr Osteoporsis Rep.

